# Magnitude of placebo response in clinical trials of paroxetine for vasomotor symptoms: a meta-analysis

**DOI:** 10.3389/fpsyt.2023.1204163

**Published:** 2023-08-04

**Authors:** Joshua R. Rhodes, Cameron T. Alldredge, Gary R. Elkins

**Affiliations:** ^1^Department of Psychology, Abilene Christian University, Abilene, TX, United States; ^2^Department of Psychology and Neuroscience, Baylor University, Waco, TX, United States

**Keywords:** placebo response, hot flashes, paroxetine, meta-analysis, vasomotor symptoms

## Abstract

**Introduction:**

Vasomotor symptoms, or hot flashes, are among the most common complaints for menopausal and postmenopausal women. As an alternative to hormone replacement therapy, paroxetine mesylate became the only non-hormonal treatment approved by the U.S. Food and Drug Administration (FDA), despite limited evidence for its efficacy. More specifically, there is uncertainty around paroxetine's unique benefit and the magnitude of the placebo response in clinical trials of paroxetine.

**Methods:**

Relevant databases were searched to identify randomized clinical trials examining the efficacy of paroxetine to treat hot flashes. The primary outcomes of interest were hot flash frequency and hot flash severity scores. Data was extracted from the published results, and risk of bias assessments were conducted.

**Results:**

Six randomized clinical trials that included a total of 1,486 women were coded and analyzed. The results demonstrated that 79% of the mean treatment response for hot flash frequency is accounted for by a placebo response, resulting in a mean true drug effect of 21% at most. Additionally, 68% of the mean treatment response for hot flash severity is accounted for by a placebo response, resulting in a maximum true drug effect of 32%.

**Discussion:**

The results herein call into question the actual efficacy of the only FDA approved, non-hormonal treatment for hot flashes by demonstrating that a placebo response accounts for the majority of treatment responses for reductions in both hot flash frequency and severity. The findings provide evidence to reevaluate the use of paroxetine to treat postmenopausal hot flashes and emphasize the importance of considering effective, alternative treatments for vasomotor symptoms.

## 1. Introduction

Menopause, as defined by a 12-month period of amenorrhea following the final menstrual period, is a natural, biological process that occurs in women across the world. While this transitional period varies between individuals, the median age of onset is 51 years and is characterized by ovarian follicular depletion and a reduction in ovarian estrogen secretion (1). In some circumstances, the menopausal transition may be surgically induced as a result of a bilateral oophorectomy, or medically induced in populations such as cancer patients undergoing chemotherapy. Various symptoms are associated with menopause, the most well-known being the presence of vasomotor symptoms (VMS; i.e., hot flashes or hot flushes) which occur in over 75% of menopausal women and over 50% in breast cancer survivors (2–4). Hot flashes are characterized by elevated skin temperature and blood flow in areas such as cheeks, forehead, chest, fingers, and toes often co-occurring with palpitation, sweating, and anxiousness (5–7). A longitudinal analysis in 2006 found that up to 80% of women surveyed during the menopausal transition reported experiencing VMS within a two-week interval, with the greatest frequency occurring during the transition from early to late menopause (8). The Study of Women's Health Across the Nation (SWAN) found that despite the commonly held belief that VMS only last a few years, women reported frequent hot flashes and night sweats for a median of 7.4 years, with many cases lasting much longer (9, 10).

While the underlying mechanisms of hot flashes remain unclear, most theories involve changes in the impact of serotonin and norepinephrine levels on the body's thermoregulatory process and in levels of reproductive hormones (11, 12). The thermoregulatory hypothesis centers around the *thermoregulatory zone*, a homeostatic range of core body temperature. Fluctuation of core body temperature above or below this range triggers physiological responses in the body such as sweating when it exceeds the upper threshold and chills when below the threshold. This thermoregulatory zone between sweating and shivering is referred to as the “thermoregulatory null zone” and is sensitive to a 0.4°C temperature fluctuation (13). Women with VMS experience a disruption in this neuroendocrine and autonomic thermoregulatory process, specifically a hypothesized narrowing of this zone. In this, VMS is characterized as an exaggerated response to these disruptions, resulting in an exacerbated sweating or shivering response (14–17).

The decline in estrogen production is a guaranteed symptom of the menopausal transition and estrogen levels within the body have historically been linked with the occurrence of hot flashes. Research indicates that it is the withdrawal of estrogen that leads to VMS supported by findings that women with gonadal dysgenesis, atypical gonadal development resulting in low levels of estrogen, generally do not experience VMS unless they undergo estrogen replacement therapy that is later ceased. Additionally, women who undergo medical procedures such as an oophorectomy, resulting in the sudden withdrawal of estrogen, experience a rapid occurrence of hot flashes (18, 19). The alteration in estrogen levels may also indirectly affect the thermoregulatory zone through its impact on levels of central nervous system neurotransmitters, serotonin and norepinephrine.

Additional complexity is introduced by research findings which indicate there is some level of racial and ethnic variations in the experience of VMS, with Western countries tending to report higher prevalence rates of VMS than others, such as Asian countries (20). Data also suggests that Black women have the highest prevalence, longest duration, and experience the most difficulties from VMS (10, 8; (21)). Avis et al. (10) also found that independent of race/ethnicity, women in low socioeconomic groups were more likely to experience VMS. What does not remain unclear, however, is the deleterious impact of VMS on an individual's day-to-day life, including problems with sleep, depressive symptoms, anxiety, cognitive performance, and sexual health (9). More specifically, past research has suggested that compared to women who don't experience hot flashes, women with VMS experienced 66% more fatigue, 63% poorer quality of sleep, and 20% poorer physical health (22).

The lack of clarity surrounding the etiology of VMS in menopause contributes to the lack of consensus regarding the optimal treatment intervention. A widely utilized and historically effective treatment for hot flashes is hormone (estrogen) replacement therapy (HRT). While effective, HRT carries high risk as numerous studies have linked this treatment to higher incidence rates of breast cancer, coronary heart disease, pulmonary embolism, and stroke, resulting in its general contraindication for breast cancer survivors (23–25). Additional treatments include progestational agents; neuroactive agents such as clonidine, selective serotonin reuptake inhibitors (SSRI), selective norepinephrine reuptake inhibitors (SNRI), and gabapentin; alternative remedies such as black cohosh, ginseng, and dong quai; and behavioral interventions such as yoga, hypnosis, exercise, and relaxation techniques (1, 26, 27). Evidence for efficacy varies among these treatments as many have not consistently shown improvements greater than placebo effect. However, hypnosis as a VMS treatment seems to exhibit the most data-driven efficacy, with two studies resulting in a 69% reduction in hot flashes from baseline to endpoint (27, 28) and an additional 12-week clinical trial resulting in a significant reduction of hot flash frequency by 74% compared to a reduction of 17% by the active structured attention control treatment (29). While a well-defined hypnosis intervention is a promising and potentially efficacious VMS treatment, the specific mechanism of action in the reduction of hot flashes is unknown.

The need for a widely dispersed, non-hormonal treatment more effective than placebo has been a focus of research. This has led to a focus on paroxetine mesylate, an SSRI traditionally used in the treatment of depression (7, 30). As a result of this research, Brisdelle^TM^ (paroxetine mesylate 7.5 mg/d) is the first and only FDA-approved, non-hormonal treatment for VMS, gaining approval in 2013 (31). This is problematic for two reasons: [1] the researchers reported that the improvement was modest and likely had questionable clinical significance and [2] the FDA Reproductive Health Drugs Advisory Committee recommended *against* the approval of Brisdelle^TM^, indicated by a vote of 10 to 4, concluding that the drug's benefit-risk profile was not satisfactory for approval (32).

Brisdelle^TM^ (paroxetine mesylate) is a selective serotonin reuptake inhibitor (SSRI) with a strength of 7.5 milligrams (mg), to be taken orally once per day. The 7.5 milligrams per day (mg/d) dosage of Brisdelle^TM^ is notably lower than the paroxetine dosage used as an antidepressant (starting at 20 mg/d). Proposed mechanistic theories for SSRI's on VMS have included their ability to decrease blood flow to the individual's skin in order to counteract the vasodilation that one experiences during hot flashes, and the lowering of the individual's core body temperature through central vasodilation (33). While unproven and unclear, paroxetine's mechanism has been proposed to be mediated by the activation of serotonin receptors in the hypothalamus (34). This mechanism of action is directly related to the theorized *thermoregulatory dysfunction* caused by changing levels of serotonin and norepinephrine (35). The prescribing information of Brisdelle^TM^ reports common adverse events to include headache, fatigue, and nausea/vomiting with a specific warning for increased risk of suicidal ideation in pediatric, adolescent, and adult use (36, 37).

The uncertainty regarding the mechanism of action of paroxetine leads to an increased focus on examining paroxetine's efficacy in treating VMS. Typically, when determining efficacy, experimental drugs are analyzed in comparison to a placebo. When an experimental drug is found to be significantly more effective than a placebo, the placebo response is often dismissed and rarely analyzed, regardless of its magnitude. This common disregard for the placebo response after data analysis has led to various studies examining the magnitude of the placebo response in common, well accepted pharmacological treatments. As the understanding of placebos has grown, we now know that a placebo is more than simply an inert treatment and involves the whole ritual of the therapeutic act itself which gives important insight into the potential mechanisms behind the healing process (38). A major psychological theory around the efficacy of placebos is cognitive expectancy.

Cognitive expectancy has been demonstrated to be influenced by a variety of factors such as medication brand name, apparent dose, mode of administration, condition being treated, and even the color of the placebo itself (39). These variations in the placebo are coupled with variations in intuitive expectancies regarding their effectiveness. The construct of expectancy can be further separated into the difference between *response expectancies* and *stimulus expectancies*. Response expectancies are operationalized as an individual's predictions of their own nonvolitional responses to events, whereas stimulus expectancies are their anticipations of external events (40, 41).

In a groundbreaking meta-analysis, Kirsch and Sapirstein (42) analyzed the magnitude of the placebo response in the use of antidepressant medications for depression, finding that ~75% of the response to active antidepressant medication is due to the response to inert placebos. These highly debated results have been consistently replicated in studies finding that the difference in effect between antidepressants and placebos on the Hamilton Depression Rating Scale (HAM-D, 42) is consistently below the threshold for clinical significance (43–47). Notable responses to placebo have also been found in symptoms such as pain, anxiety, IBS, and erectile dysfunction (48–51).

Comprehensive literature regarding the magnitude of the placebo response for VMS is scarce, although overviews of previous trials provide insight that a substantial placebo response exists in the treatment of hot flashes (52). One quantitative analysis found that in addition to placebo responses being higher in trials of hormonal drugs, the placebo response for VMS increased over time, reaching a plateau after approximately the 12th week of treatment (53). As previously noted, these high rates of placebo response can be due to a variety of factors. One factor that is unique to studies for the management of hot flashes is the use of a hot flash daily diary in which participants continually monitor and self-report hot flashes, a practice that, by itself, may play a role in the reduction of VMS.

Keeping in mind (1) the scant evidence of clinical significance in the use of SSRIs for depression, (2) high rates of placebo response in the use of SSRIs among other common treatments, and (3) the limited efficacy so far exhibited by paroxetine mesylate for VMS, this meta-analysis aims to analyze and quantify the magnitude of the placebo response in clinical trials using paroxetine mesylate for the treatment of VMS. Findings of any degree will have implications for the treatment of VMS moving forward. If truly and uniquely effective, paroxetine mesylate offers a welcome alternative to HRT as its use mitigates the serious health risks associated with estrogen therapy. Conversely, if the active pharmacological effect of paroxetine mesylate is not substantially effective, then millions of women could be unnecessarily experiencing adverse side effects due to a medication that essentially functions as an active placebo.

## 2. Methods

### 2.1. Eligibility citeria

Studies included in this meta-analysis were chosen according to the following eligibility criteria: (1) participants experiencing VMS, regardless of history of cancer; (2) randomized, placebo-controlled trials comparing paroxetine of any dosage against placebo; (3) outcome measure of average hot flash frequency (daily or weekly); (4) sufficient data reported to calculate within-group effect sizes (for treatment and control groups); and (5) publication in the English language.

### 2.2. Information sources and search methods

A comprehensive literature search was conducted using electronic databases (PubMed, psychINFO, *ClinicalTrials.gov*) and scanning reference lists of articles in the specific field of study. Search terms used were various combinations of the following controlled terms: “paroxetine,” “brisdelle,” “hot flash,” “hot flush,” and “vasomotor.” There were no limitations applied to the date of publication. The last search was run on 01 November 2020.

### 2.3. Study identification

Previously mentioned electronic databases were searched by a single independent reviewer. Titles and abstracts were reviewed in order to determine which studies met a priori eligibility criteria. If the abstract did not contain sufficient information, the full-text manuscript was obtained for a final determination of eligibility status. Once a study was determined to be eligible, full-text manuscripts were obtained for assessment and data extraction.

### 2.4. Data collection and extraction

One review author extracted pre-specified data from included studies and a second reviewer checked the extracted data. If any disagreements or discrepancies occurred in data extraction, it was planned that a third reviewer would make a final decision. Data was extracted from each included study on the following: (1) characteristics of trial participants including age and race/ethnicity; (2) inclusion and exclusion criteria for included studies; (3) study characteristics including origin, sample size, study design, study duration, and paroxetine dose administered; (4) hot flash frequency data (mean frequency at baseline and endpoint); and (5) hot flash severity data (mean severity at baseline and endpoint), when applicable.

### 2.5. Risk of bias assessment

Risk of bias was assessed using the Cochrane Risk of Bias tool for randomized trials (ROB 2). This tool is results-based in its assessment of risk of bias and assesses the bias for each specific outcome instead of an overall risk of the trial. Each possible domain for risk includes signaling questions that help assess the risk of bias for random sequence generation, allocation concealment, blinding of participants and research personnel, blinding of outcome assessment, incomplete outcome data, selective reporting, and other bias. Bias reported in these findings is in reference to both outcomes of VMS severity and frequency as they were simultaneously measured.

### 2.6. Summary of measures

The primary outcome measure to determine the magnitude of placebo response was hot flash frequency, measured by participant self-report. Hot flash frequency, in the studies included, was generally calculated as the sum of hot flashes recorded in a daily hot flash diary for seven calendar days in the specific treatment week. The exception to this is found in Simon et al. (54) studies in which hot flash frequency at baseline was calculated as follows, where *x* is the number of moderate to severe hot flashes and *n* is the number of days in the placebo run-in period:


$$\begin{array}{l} \left[\left(x\;on\;day\;1\;+\;x\;on\;day\;2\;+\;\ldots \;+\;x\;on\;day\;n\right)/\left(n\;1\right)\right]\times 7 \end{array}$$

The secondary measure assessed was hot flash severity score (hot flash composite severity score), a common secondary measure across the majority of clinical trials included. Severity scores were calculated using various methods, most commonly using assigned ranking numerical values to hot flash severity category. For example, in the clinical trials used for FDA approval (54), hot flash severity score was calculated by the following formula, where *F*_m_ and *F*_s_ represent the frequency of moderate and severe hot flashes experienced, respectively, in the identified treatment week:


$$\begin{array}{l} \left(2Fm\;+\;3Fs\right)\;÷\;\left(Fm\;+\;Fs\right) \end{array}$$

### 2.7. Statistical analysis

Traditional effect size calculation employs the use of a standardized difference score, statistic *d*. In order to calculate *d*, the mean of the control group is subtracted from the mean of the experimental group and the difference is divided by the pooled standard deviation. Kirsch and Sapirstein (42) outline that in order to calculate the effect size of a placebo, the effects of a no-placebo control group would be required. This requirement introduces complexity to the situation as placebos themselves are generally used as the control group. To ameliorate this problem, the calculation of within-cell or pre-post effect sizes were determined by the subtraction of posttreatment mean scores from pretreatment mean scores, and the difference was divided by the pooled standard deviation. Calculation of effect sizes for studies that did not provide posttreatment standard deviations (*SD*) were executed by using pretreatment *SD*s in place of pooled standard deviations. Calculation of effect size for studies that reported the standard error (*SE*) of hot flash frequency were executed by multiplying this value by the square root of the group sample size to obtain the standard deviation (*SD*) for effect size calculation. Additionally, for studies examining the effects of differing doses, a single effect size was calculated for each dosage and entered independently into the analysis. Once these calculations are completed for the placebo control group and the experimental medication group, it is now possible to estimate the proportion of the response to the medication that is duplicated by the administration of a placebo. This is done by subtracting the pre-post effect size of the placebo control group from the pre-post effect size of the treatment group, resulting in the difference representing the unique effect of the treatment group not accounted for by the administration of a placebo.

With the exception of one study (55), all included randomized trials were a parallel study design. The remaining study was a cross-over design for which only the first arm of the crossover was included in analysis. This first arm of the crossover included differing doses of paroxetine and two placebo groups. Three of the included studies (54, 56) report mean daily hot flash frequency at baseline and mean weekly reduction of hot flash frequency at endpoint. Due to this, the baseline daily average was multiplied by seven to create a baseline weekly average. The endpoint average weekly reduction was then subtracted from the baseline weekly average and the difference is referred to as the endpoint weekly average of hot flash frequency. For those studies that reported baseline daily hot flash frequency, this difference is then divided by seven to obtain an endpoint daily average hot flash frequency.

## 3. Results

### 3.1. Search results and study selection

**Figure 1:**
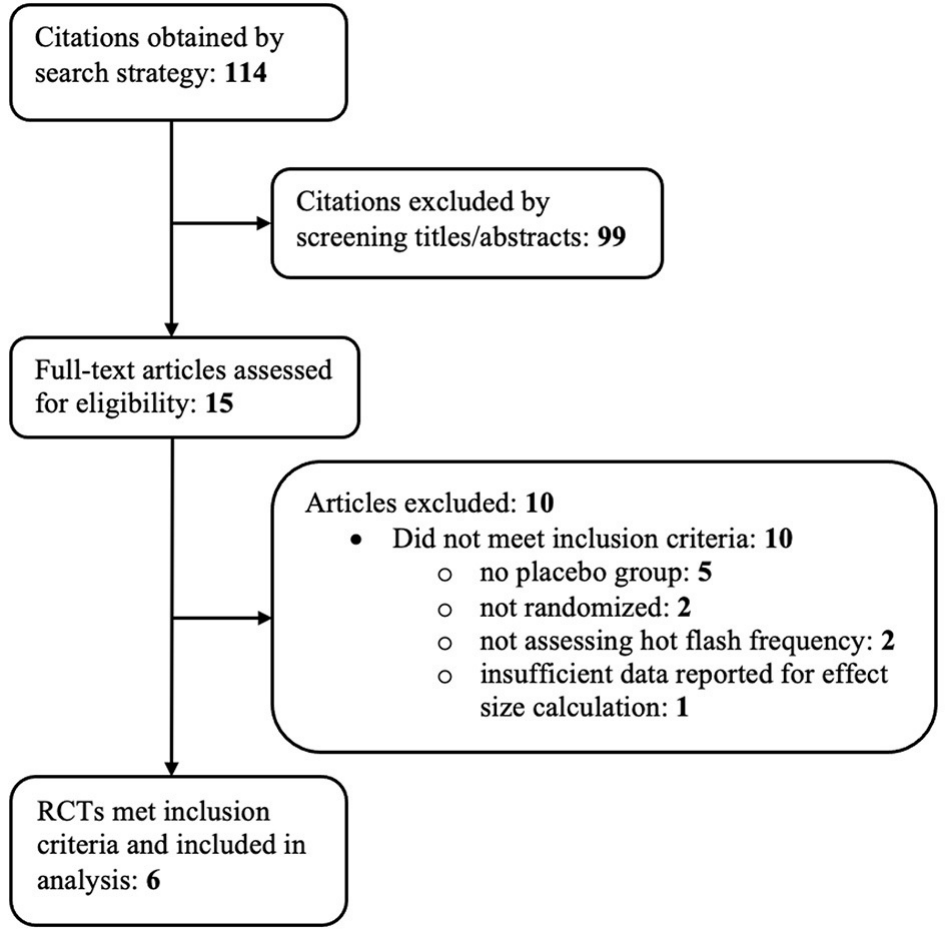
Study flow diagram.

A total of 114 references were identified through the previously mentioned electronic search strategy. Ninety-nine of these references were excluded based on examination of their titles and abstracts, leaving 15 references for which full-text manuscripts were obtained for further investigation. Ten of these articles were excluded based on inclusion criteria. One manuscript (57) met all inclusion criteria except had inadequate data reported to calculate within-group effect sizes. Figure 1 depicts the study flow diagram and includes details of reasons for exclusion of studies. Six randomized controlled trials, reported in 4 papers, met the pre-determined inclusion criteria and were included in the analysis.

The six RCTs included a total of 1,486 menopausal and post-menopausal women, in addition to women experiencing vasomotor symptoms without the specification of menopausal status. Hot flash frequency and severity were assessed for a period ranging from 8 weeks to 24 weeks. The participant age range was 36 to 76 years of age with a predominately ethnically Caucasian/White study population, when reported. African American/Black was the second most frequently reported ethnicity.

The six RCTs were reported in 4 published articles, with one article containing results of two separate trials (54). The remaining trial (58) was not published in manuscript form but was registered and data was reported to *ClinicalTrials.gov*. Table 1 further describes study characteristics of included RCTs.

**Table 1:** Studies included in analysis.

**Study**	**Study design**	**Treatment period**	**Study arms: number of subjects per arm**	**Study population**
Stearns et al. (55)	Stratified, randomized, double-blind, cross-over, placebo-controlled	8-Week	Paroxetine 10 mg/d: 37 Paroxetine 20 mg/d: 38 Placebo: 76	Women experiencing ≥ 2 hot flashes per day for 1 month or longer prior to enrollment
Simon et al. (54) Study #1	Multi-center, double-blind, placebo-controlled, phase 3 study	12-Week	Paroxetine 7.5 mg/d: 301 Placebo: 305	Menopausal females > 40 years experiencing ≥ 7 moderate to severe hot flashes per day before screening
Simon et al. (54) Study #2	Multi-center, double-blind, placebo-controlled, phase 3 study	24-Week	Paroxetine 7.5 mg/d: 284 Placebo: 284	Menopausal females >40 years experiencing ≥7 moderate to severe hot flashes per day before screening
Simon et al. (59)	Randomized, double-blind, placebo- and active-controlled	16-Week	Paroxetine 20 mg/d: 6 Raloxifene 60 mg/d: 18 Placebo: 18	Postmenopausal women ≥40 years experiencing ≥5 hot flashes per week
Capriglione et al. (56)	Randomized, double-blind, placebo-controlled	16-Week	Paroxetine 7.5 mg/d: 42 Placebo: 38	Postmenopausal, gynecological cancer survivors
Noven Therapeutics (58)	Multicenter, randomized, double-blind, placebo-controlled	8-Week	Paroxetine 7.5 mg/d: 49 Placebo: 52	Menopausal women ≥40 years experiencing and average of ≥7 moderate to severe hot flashes per day

### 3.2. Risk of bias within studies

**Figure 2:**
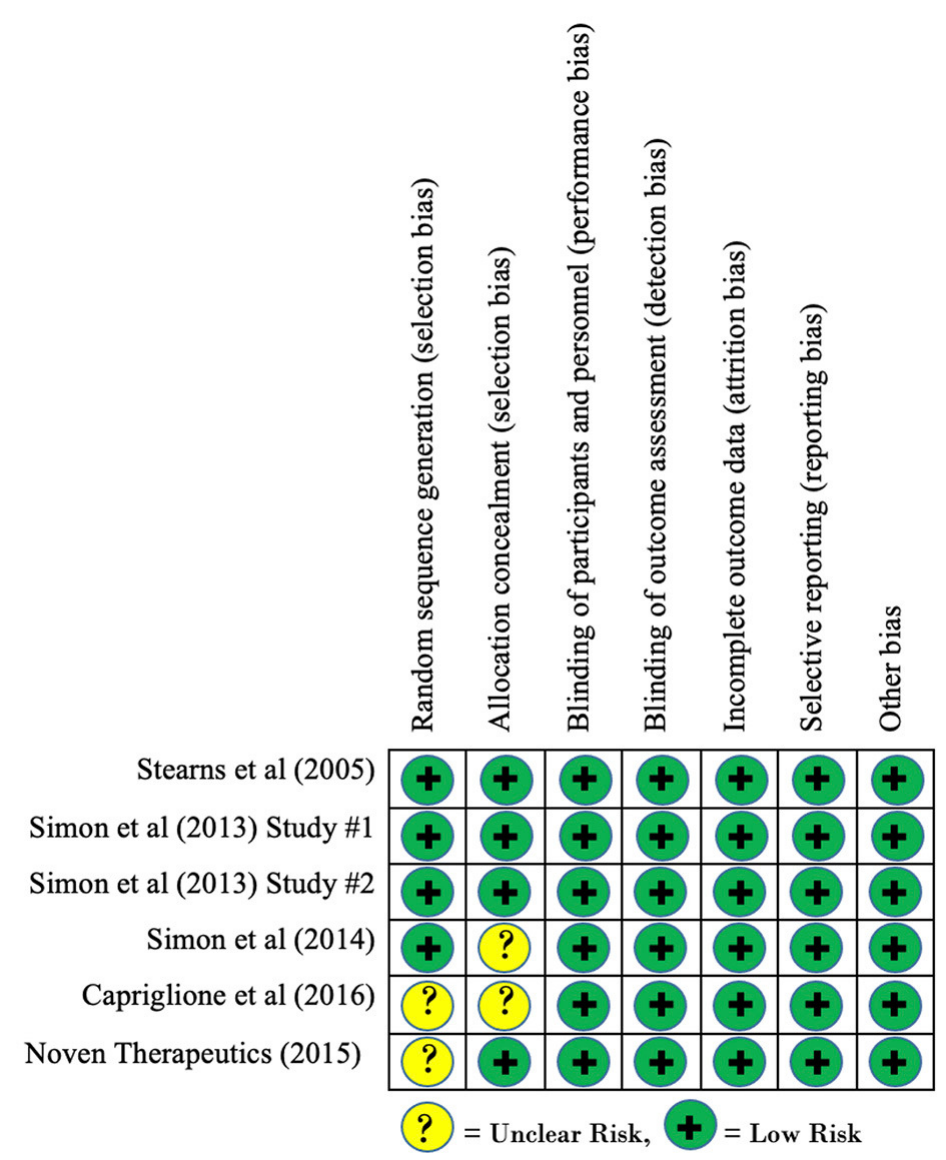
Risk of bias within studies.

Risk of bias of included clinical trials, as measured using the Cochrane Risk of Bias tool, is reported in Figure 2. Overall, no clinical trials were found to contain any domains that demonstrated a high risk of bias. Three studies demonstrated an unclear risk of bias for the domain of allocation concealment as the published findings did not provide enough information to make a determination. Similarly, two studies demonstrated an unclear risk of bias for the domain of random sequence generation as inadequate information was provided regarding sequence generation. Based on these findings, there is no concern for risk of bias within studies to have a significant effect on our findings for within-group effect sizes.

### 3.3. Percentage reduction across studies

The first result of interest, especially for individuals considering using paroxetine as a VMS treatment, is a simple examination of average percentage reduction of symptomatology across studies. Average percentage reduction for both paroxetine and placebo was calculated for each study. These averages were then combined and weighted by sample size to determine the weighted average percentage reduction of hot flash frequency across all studies included. Calculations indicate that paroxetine reduced hot flashes by 51% on average (−5.67 hot flashes per day) with placebo reducing hot flashes by 39% on average (−4.34 hot flashes per day). These results indicate that based on a simple inspection of frequencies, the pharmaceutical effect of paroxetine seemed to decrease hot flashes by an additional 12% than placebo. In its simplest form, this means that on average, individuals who were administered paroxetine experienced an additional reduction of just over one hot flash per day compared to those who received a placebo. While this inspection of frequencies is informative, more critical information is gained from the calculation of effect sizes for each treatment group.

### 3.4. Effect size results of individual studies

The only non-parallel study design was a four-arm crossover study design in which researchers examined differing doses of paroxetine (10 mg/d and 20 mg/d) against two placebo groups designed to match the 10 mg and 20 mg group (55). Both phases of the study consisted of 4 intervention weeks; however, only phase 1 data was included in this analysis to avoid any effect from treatment cross-over. Results from this study report a significantly greater reduction in hot flash frequency and severity for both treatment arms. Our analysis is congruent with these results regarding frequency, finding a drug response effect size (*d*) of 0.91 and 0.76 for the paroxetine 10 mg and 20 mg groups, respectively, and a placebo response effect size (*d*) of 0.30 and 0.35 for the 10 mg and 20 mg placebo groups. These findings indicate that for hot flash frequency, approximately only 33% of the drug response can be accounted for by the exhibited placebo response in the 10 mg group and ~46% can be accounted for by the placebo response in the 20mg group. Concerning hot flash severity, researchers assessed this variable through the measurement of a daily composite severity score which was calculated by multiplying the number of corresponding hot flash severity (mild, moderate, severe, or very severe) by their respective assigned values of 1, 2, 3, or 4. These values were added together to generate a summary score. Effect size analysis for hot flash severity indicates a drug response effect size (*d*) of 0.74 and 0.81 for the 10 mg and 20 mg groups, respectively, and a placebo response effect size (*d*) of 0.32 and 0.33 for the 10 mg and 20 mg placebo groups. These findings indicate that for hot flash severity, the placebo response accounts for ~43% of the exhibited drug response in the 10 mg group and 41% in the 20mg group.

The first of the two studies leading to the FDA approval of paroxetine mesylate 7.5 mg was a 12-week, Phase III clinical trial evaluating paroxetine mesylate 7.5 mg against placebo for moderate-to-severe VMS, with hot flash frequency and severity being the primary outcomes measured (54). Results from this 12-week study show a greater mean weekly reduction in VMS daily frequency for the paroxetine 7.5 mg group than for the placebo group at endpoint (−43.5 and −37.3, respectively; *p* = 0.009). While this difference in mean weekly reduction at endpoint is statistically significant, our analysis finds a drug response effect size (*d*) of 1.28 and a placebo response effect size (*d*) of 1.21, indicating that ~95% of the drug response can be accounted for by the exhibited placebo response. Similarly, the analysis found a drug response effect size (*d*) of 0.33 and a placebo response effect size (*d*) of 0.29 for change in hot flash severity, indicating that ~88% of the change in hot flash severity was accounted for by the exhibited placebo response. The method used to calculate hot flash severity for both studies used during FDA approval has been previously discussed.

The second study used for FDA approval is a 24-week, Phase III clinical trial also evaluating paroxetine mesylate 7.5 mg against placebo for moderate-to-severe VMS (54). While the duration of the study was 24 weeks, treatment endpoint used for data analysis by investigators was at 12 weeks, with the 24-week endpoint used as an additional efficacy endpoint to examine persistence of treatment benefit. Results from this study also show a superior mean weekly reduction in VMS frequency for the paroxetine mesylate 7.5 mg group compared to placebo at 12 weeks (-37.2 and−27.6, respectively; *p* = 0.0001). While this difference in mean weekly reduction at endpoint is also statistically significant, our analysis finds a drug response effect size (*d*) of 1.38 and a placebo response effect size (*d*) of 0.99, indicating that ~72% of the drug response can be accounted for by the exhibited placebo response. The analysis for hot flash severity found a drug response effect size (*d*) of 0.40 and a placebo response effect size (*d*) of 0.22, indicating that ~55% of the drug response can be accounted for by the exhibited placebo response.

A 12-week study examined paroxetine (20 mg/d) against non-active placebo and active-control group (raloxifene 60 mg/d) (59). This study examined groups with very small sample sizes, inhibiting the ability for investigators to detect significant changes between groups. Both hot flash frequency and severity were measured, with a hot flash severity score being calculated as follows:


$$\begin{array}{l} \;1\;(number\;of\;weekly\;mild\;hot\;flashes)\\ +\;2\;(number\;of\;weekly\;moderate\;hot\;flashes)\\ \;+\;3\;(number\;of\;weekly\;severe\;hot\;flashes)\\ +\;4\;(number\;of\;weekly\;very\;severe\;hot\;flashes) \end{array}$$

Results from this study indicate no significant difference in hot flash frequency between paroxetine (20 mg/d) and placebo at endpoint; however, paroxetine had a greater numerical reduction of hot flash frequency. Conversely, the placebo group was the only group with a significant reduction of hot flash severity at endpoint with a greater numerical reduction in hot flash severity than the paroxetine group. It should be of note that in addition to small sample sizes limiting ability to detect significant differences between groups, it also influences effect size calculation within groups dues to dramatic differences in standard deviations at baseline between groups. Keeping the difference in standard deviation in mind, the effect size analysis for hot flash frequency indicates a drug response effect size (*d*) of 0.63 and a placebo response effect size (*d*) of 0.92. Similarly, the effect size analysis for hot flash severity indicates a drug response effect size (*d*) of 0.38 and a placebo response effect size (*d*) of 0.81. These analyses indicate that for hot flash frequency, the placebo response effect size was ~1.5 times greater than the drug response effect size. Regarding hot flash severity, the analysis indicates that the placebo response effect size was ~2 times greater than the drug response effect size.

A more recent study conducted in 2016 was a 16-week clinical trial examining the effects of paroxetine 7.5 mg against placebo in gynecological cancer survivors (56). Results from this study indicate a significantly greater reduction in mean weekly VMS frequency for paroxetine 7.5 mg than for placebo at week 16 (−46.5 and −39.3, respectively; *p* = 0.009). While a significant difference exists for mean VMS frequency reduction, our analysis finds a drug response effect size (*d*) of 1.94 and a placebo response effect size (*d*) of 1.74, indicating that ~90% of the drug response can be accounted for by the exhibited placebo response. Although it was an included study measure, the published manuscript for this clinical trial failed to report baseline and endpoint VMS severity, therefore it is not feasible to calculate pre-post effect sizes for either study arm. Despite not reporting baseline and endpoint (week 16) VMS severity, the authors do report a significant difference in mean weekly reductions from baseline to week 4, with the paroxetine group being superior (−0.09 and −0.05; *p* = 0.0048).

The most recent study being the eight-week efficacy and safety study performed by Noven Therapeutics examined the effect of paroxetine mesylate 7.5 mg against placebo (58). Results from this study indicate greater reduction in mean weekly VMS frequency for the paroxetine 7.5 mg group than for placebo, although the difference was not significant (−42.2 and −35.5, respectively; *p* = 0.0541). Our analysis finds a drug response effect size (*d*) of 1.92 and a placebo response effect size (*d*) of 1.34, indicating that ~70% of the drug response can be accounted for by the exhibited placebo response. Hot flash severity scores were calculated for each participant using the following formula, with Fm representing the frequency of moderate hot flashes and Fs representing the frequency of severe hot flashes experienced in the designated treatment week:


$$\begin{array}{l} \left(2\times Fm\;+\;3\times Fs\right)\;÷\;\left(Fm\;+\;Fs\right) \end{array}$$

Results indicate a significant difference in favor of the paroxetine group for mean change from baseline in hot flash severity (−0.133 and −0.066, *p* = 0.0364). Effect size analysis for hot flash severity finds a drug response effect size (*d*) of 0.44 and a placebo response effect size (*d*) of 0.26, indicating that ~59% of the drug response can be accounted for by the exhibited placebo response.

### 3.5. Synthesis of effect size results

The summary of sample size and effect size for hot flash frequency in included studies is found in Table 2. Results of effect size calculations indicate that the mean effect size across treatment (paroxetine) groups for hot flash frequency, weighted for sample size, was 1.35 *SD*s. Additionally, the mean effect size across placebo groups for hot flash frequency, weighted for sample size, was 1.07 *SD*s. Kirsch and Sapirstein (42) found that if there are no significant pretreatment between-group differences, the difference between pre-post effect sizes of the treatment and control group is equivalent to the conventional effect size calculation. Therefore, the difference between these within-group effect sizes for hot flash frequency indicates a mean unique treatment effect size of .28 *SD*s. These findings indicate that across all studies included in analysis, ~79.26% of the response to paroxetine for hot flash frequency is accounted for by a placebo response, resulting in a mean true drug effect of 20.74% at most.

**Table 2:** Sample and effect size for hot flash frequency for included studies.

	**Paroxetine**		**Placebo**	
**Study**	***n***	***d***	***n***	***d***
Stearns et al. (55) (10 mg)	37	0.91	39	0.30
Stearns et al. (55) (20 mg)	38	0.76	37	0.35
Simon et al. (54) (12 Week)	301	1.28	305	1.21
Simon et al. (54) (24 Week)	284	1.38	284	0.99
Simon et al. (59)	6	0.63	18	0.92
Capriglione et al. (56)	42	1.94	38	1.74
Noven (2015)	49	1.92	52	1.34

The summary of sample size and effect size for hot flash severity in included studies is found in Table 3. Results of effect size calculation indicate that the mean effect size across treatment (paroxetine) groups for hot flash severity, weighted for sample size, was 0.41 *SD*s. Additionally, the mean effect size across placebo groups for hot flash severity, weighted for sample size, was .28 *SD*s. Using the same precedent as before, the difference between these within-group effect sizes for hot flash severity indicates a mean unique treatment effect size of 0.13 *SD*s. These findings indicate that across all studies included in analysis, ~68.29% of the response to paroxetine for hot flash severity is accounted for by a placebo response, resulting in a mean true drug effect of 31.71% at most.

**Table 3:** Sample and effect size for hot flash severity for included studies.

	**Paroxetine**		**Placebo**	
**Study**	***n***	***d***	***n***	***d***
Stearns et al. (55) *(10mg)*	37	0.74	39	0.32
Stearns et al. (55) *(20mg)*	38	0.81	37	0.33
Simon et al. (54) *(12 Week)*	301	0.33	305	0.29
Simon et al. (54) *(24 Week)*	284	0.40	284	0.22
Simon et al. (59)	6	0.38	18	0.81
Noven (2015)	48	0.44	51	0.26

## 4. Discussion

These findings indicate that for the average individual taking paroxetine, ~79% of their experienced reduction in hot flashes would also have been achieved through the administration of an inert placebo. Previous meta-analyses have noted this modest treatment benefit when paroxetine is compared to placebo, specifically in the two clinical trials used for FDA approval (60). Despite minor treatment benefit and a vote against its approval by the U.S. FDA Advisory Committee for Reproductive Health Drugs panel, paroxetine mesylate 7.5 mg was eventually approved by the FDA for treatment of moderate-to-severe VMS. It is important to note that these findings do not reflect the percentage of individuals who may benefit from paroxetine treatment, rather the percentage of the reduction in frequency that is accounted for by the placebo response.

It can be hypothesized that paroxetine's unique treatment effect is a result of its specific pharmacological effect in the reduction of hot flashes; however, without fully understanding its mechanisms, we cannot definitively know. The lack of clarity regarding the mechanisms by which paroxetine has an effect on hot flashes has not been fully determined by research. The limited understanding regarding the mechanism of clinical action of common antidepressants has resulted in difficulty identifying their specific pharmacological effects for depression. In the same light, once the effect size of the placebo response has been accounted for in the included clinical trials, it is still not feasible to make the claim that the resulting effect is solely due to the pharmacological mechanisms of paroxetine. One possibility for this difference in efficacy, although admittedly controversial, is that paroxetine may act as an active placebo in the treatment of vasomotor symptoms. Active placebos, while still acting as a placebo in clinical trials, are by their very definition distinct from inert (or inactive) placebos in that they are active medications without a documented specific activity for the targeted condition or symptom. The utility of active placebos is their pharmacological generation of side effects, a subset of side effects distinct from those experienced by individuals only administered an inactive placebo.

As pharmacological advancements have been made, they have routinely been coupled with the expectation of side effects to some degree. The expectation of side effects plays a critical role in clinical trials for pharmacological substances for many reasons, two of which are important to discuss in light of these findings. First, in blinded clinical trials involving the administration of inactive placebos, the expectation of side effects can elicit those side effects even in participants randomized to the placebo group. Second, in clinical trials involving the administration of active placebos, the pharmacologically induced side effects of these active placebos can lead participants to conclude that they are in the active medication arm of the study simply due to the experience of side effects. This conclusion can spark the generation of even greater expectancies of symptom reduction. It is important to note that the complex interplay between placebos and side effects, both pharmacologically induced and not, is interwoven throughout the entire construction of clinical trials and must be analyzed in an attempt to avoid letting this relationship muddy the proverbial waters of the study drug's pharmacological effect.

This knowledge of the role of expectancy in the placebo effect provides evidence for the previous claim of paroxetine's role as an active placebo in VMS treatment. When participants in these inactive placebo clinical trials have the expectation of side effects, their experience of them, or lack thereof, can lead to the assumption of being randomized to the corresponding study group. This may result in both an enhanced placebo effect in active drug groups and a weakened placebo effect in placebo groups. Evidence of this relationship can be observed in findings from a meta-analysis of fluoxetine that reported a correlation of 0.85 between the therapeutic effect of the drug and the percentage of patients reporting side effects (61). In summary, the ability of participants to correctly identify their assigned treatment arm through the experience of side effects may serve as a mechanism of enhancement for an overall placebo effect of paroxetine that has been misidentified as the apparent drug effect.

While this analysis is able to identify the magnitude of placebo response, it is unable to identify the actual placebo effect across clinical trials. As previously discussed, a placebo response includes changes that could also have occurred without giving the participant a placebo. In order to analyze the placebo effect, it is necessary to also identify the natural history effects that occur in a no-treatment or wait-list control group. This information would allow one to conclude what proportion of the exhibited placebo response is a result of generated expectancies from placebo administration, eliminating the variance accounted for by what would have happened regardless of the placebo administration.

Individual study results were fairly consistent; however, three findings for hot flash frequency stand out as noteworthy. First, the Stearns et al. (55) clinical trial exhibits by far the strongest unique drug effect size in the 10 mg group (*d* = 0.61), with the placebo response only accounting for 33% of the exhibited treatment response. This magnitude of placebo response is much closer to the expected magnitude of placebo response found for an FDA approved drug, yet it stands alone in the included trials. At face value, these results provide evidence for paroxetine's efficacy; however, the remaining trials analyzed tell a different story. It may be that this trial contains unique qualities that increased the efficacy of paroxetine on VMS frequency. Stearns et al. (55) was the only cross-over trial, yet only data from the first phase was used, and had the lowest threshold for hot flash frequency inclusion criteria. Despite these qualities, it remains unknown why this trial stands out regarding hot flash frequency and severity effect size, but it speaks to the importance of needing multiple, well-designed clinical trials to determine replicability and efficacy of novel treatments. The second noteworthy finding regarding hot flash frequency is found in the incredible magnitude of placebo response found in the Simon et al. (54) 12-week study. Results from this study indicate that ~95% of the exhibited drug response was accounted for by placebo response. What makes this finding so noteworthy is that this was one of two trials leading to FDA approval of paroxetine, with the remaining trial resulting in ~72% of the drug response being accounted for by the placebo response. While one set of results does not speak for all, anytime the placebo response accounts for 95% of a treatment effect, it should seriously call into question the efficacy of the treatment, not directly lead it to approval. The third finding of interest is that results from Simon et al. (59) also stand out from the rest regarding hot flash frequency and severity. These peculiar findings can be accounted for in that very small sample sizes lead to skewed baseline standard deviations, which resulted in larger effect sizes for the placebo group than for the paroxetine group, despite a minor numerical advantage for paroxetine in the reduction of hot flash frequency.

It is at this point that there is enough data to perform a risk-benefit analysis to determine whether or not paroxetine is a worthwhile treatment option for VMS symptoms. While this decision is subjective and influenced by many factors, common benefits are that paroxetine is an FDA approved treatment that provides an alternative to hormone replacement therapy and provides a pharmacological treatment for those who are seeking one. Additionally, based on the data across studies, the individual can also expect to see a benefit of an average hot flash frequency reduction by 51% which is rightfully the most desirable aspect of using paroxetine for VMS treatment. These benefits are now held in comparison to the potential adverse side effects of paroxetine. Potential adverse effects common to the use of SSRIs include changes in mood, serotonin syndrome, akathisia (a sense of restlessness and inability to sit still), bone fracture, and its contraindications for other treatments such as monoamine oxidase inhibitors, thioridazine, and pimozide (35). Despite the low dosage of paroxetine (7.5 mg) and the administration to an older population, the treatment still carries the warning for mood changes. Additionally, paroxetine has been found to reduce the efficacy of tamoxifen for breast cancer treatment. The mechanism by which this occurs is that paroxetine is found to inhibit CYP2D6, which is an enzyme that converts tamoxifen to active metabolites (57). This specific contraindication is very concerning as breast cancer survivors may be more likely to experience more moderate to severe hot flashes as the onset of menopause in this population can be very abrupt. Overall, the use of paroxetine should co-occur with the monitoring of these potential adverse events and should avoid the concomitant use monoamine oxidase inhibitors and other serotonergic medications.

The final point of discussion is that one should take serious note of the fact that the administration of a sugar pill (placebo) accounted for 79% of the effect had by paroxetine. Because placebos are not identified as a treatment for hot flashes, the only reasonable conclusion is that there exist mechanisms at work in the placebo effect that have a significant impact on hot flash frequency and severity. The identification of these mechanisms will allow for their implementation into behavioral approaches to VMS treatment in an effort to increase efficacy with limited intervention.

Overall, these results lead to a different conclusion than previous meta-analyses conducted on the efficacy of paroxetine. Others have analyzed the difference between groups on hot flash frequency and severity reduction, concluding that paroxetine is an effective treatment for hot flashes (60, 62, 63), but have failed to assess just how much of that treatment response is accounted for by the placebo response. A significant difference between study groups is critical, but a *p*-value of less than .05 does not mean that one can disregard the effect sizes and conclude all treatment response is the result of a unique drug effect.

### 4.1. Limitations

One limitation of this meta-analysis is the limited pool of literature and data to draw from. The effect size calculations executed in this meta-analysis were from six studies, however this is also a reflection of the limited studies that originally lead to the FDA approval of paroxetine as a non-hormonal treatment for vasomotor symptoms. Despite the small group of studies analyzed, there is a sufficient patient population across included studies to draw meaningful conclusions regarding the magnitude of the placebo response in clinical trials of paroxetine for vasomotor symptoms.

As previously discussed, one included study (59) had very small sample sizes in each treatment arm, resulting in the inability to detect significant differences between groups. The inclusion of this study in effect size calculation presents a limitation as dramatic differences in baseline standard deviations influence effect size calculation. However, this potentially skewed effect size is corrected for once the weighted mean effect size is calculated for each group, using sample size as the weight. In an attempt to ensure accurate data representation, the weighted mean effect sizes were calculated for hot flash frequency and severity a second time, this time removing data from Simon et al. (59). Results from this calculation concerning hot flash frequency did not change the weighted overall effect size for the paroxetine or placebo group. Results concerning hot flash severity did not have any effect on the overall weighted effect size of the paroxetine group but did result in a decrease of the weighted effect size for the placebo group by 2/100^th^ of a point (*d* = .26). This minor decrease in placebo response effect size results in a minor shift in percentage of severity effect size accounted for by placebo response, changing the proportion from 68% to 63%.

## 5. Conclusion

This is the first meta-analysis to analyze the magnitude of placebo response in clinical trials of paroxetine for the treatment of vasomotor symptoms. The results of within-group effect size calculations have called into question the actual efficacy of the only FDA approved, non-hormonal treatment for hot flashes by demonstrating that the placebo response accounts for ~80% and 67% of the treatment response for hot flash frequency and severity, respectively. These findings have led us to four main conclusions. First, there needs to be a reevaluation of the prescription of paroxetine mesylate as a first line, nonhormonal treatment for VMS. The information regarding placebo response found in the administration of paroxetine constitutes informed consent information that may be discussed on an individual basis between patient and healthcare provider in deciding on treatment for VMS. Second, further research and consideration of paroxetine may be warranted. Third, there is a pressing need to identify more efficacious treatments for VMS. Additional research must be done to establish and provide more clinically effective alternatives to hormone therapy for VMS. Finally, these findings exhibit the need for further investigation into the mechanisms of the exhibited placebo response as a means of potentially utilizing such mechanisms in non-pharmaceutical treatments for VMS.

## Data availability statement

The original contributions presented in the study are included in the article/supplementary material, further inquiries can be directed to the corresponding author.

## Author contributions

JR and GE provided substantial contributions to the conception and design of the work, contributed to the acquisition, analysis, and interpretation of data. CA provided substantial contributions drafting the work and revising it critically for important intellectual content and verified statistical analyses and interpretations. All authors contributed to the article and approved the submitted version.

## References

[1] Morrow PKH Mattair DN Hortobagyi GN. Hot flashes: a review of pathophysiology and treatment modalities. Oncologist. (2011) 16:1658–64. 10.1634/theoncologist.2011-0174 22042786 PMC3233302

[2] Kronenberg F. Hot flashes: epidemiology and physiology. Ann N Y Acad Sci. (1990) 592:52–86. 10.1111/j.1749-6632.1990.tb30316.x 2197954

[3] Couzi RJ Helzlsouer KJ Fetting JH. Prevalence of menopausal symptoms among women with a history of breast cancer and attitudes toward estrogen replacement therapy. J Clin Oncol. (1995) 13:2737–44. 10.1200/JCO.1311.2737 7595732

[4] Chang HY Jotwani AC Lai YH Jensen MP Syrjala KL Fann JR . Hot flashes in breast cancer survivors: Frequency, severity and impact. Breast. (2016) 27:116–21. 10.1016/j.breast.02013 27065357 PMC5893329

[5] Freedman RR. Menopausal hot flashes: Mechanisms, endocrinology, treatment. J Ster Biochem Mol Biol. (2014) 142:115–20. 10.1016/j.jsbmb.08010 24012626 PMC4612529

[6] Freedman RR. Biochemical, metabolic, and vascular mechanisms in menopausal hot flashes. Fertil Steril. (1998) 70:332–7. 9696230 10.1016/s0015-0282(98)00137-x

[7] Shams T Firwana B Habib F Alshahrani A AlNouh B Murad MH . SSRIs for hot flashes: a systematic review and meta-analysis of randomized trials. J Gen Intern Med. (2014) 29:204–13. 10.1007/s11606-013-2535-9 23888328 PMC3889979

[8] Gold EB Colvin A Avis N Bromberger J Greendale GA Powell L . Longitudinal analysis of the association between vasomotor symptoms and race/ethnicity across the menopausal transition: Study of women's health across the nation. Am J Public Health. (2006) 96:1226–35. 10.2105/AJPH.2005.066936 16735636 PMC1483882

[9] El Khoudary SR Greendale G Crawford SL Avis NE Brooks MM Thurston RC . The menopause transition and women's health at midlife: a progress report from the study of women's health across the nation (SWAN). Menopause. (2019) 26:1213–27. 10.1097/GME.0000000000001424 31568098 PMC6784846

[10] Avis NE Crawford SL Greendale G Bromberger JT Everson-Rose SA Gold EB . Duration of menopausal vasomotor symptoms over the menopause transition. JAMA Intern Med. (2015) 175:531–9. 10.1001/jamainternmed.2014.8063 25686030 PMC4433164

[11] Freedman RR. Physiology of hot flashes. Am J Human Biol. (2001) 13:453–64. 10.1002/ajhb.1077 11400216

[12] Pachman DR Jones JM Loprinzi CL. Management of menopause-associated vasomotor symptoms: current treatment options, challenges and future directions. Int J Women's Health. (2010) 2:123–35. 10.2147/ijwh.s7721 21072305 PMC2971731

[13] Fisher WI Johnson AK Elkins GR Otte JL Burns DS Yu M . Risk factors, pathophysiology, and treatment of hot flashes in cancer. CA Cancer J Clin. (2013) 63:167–92. 10.3322/caac.21171 23355109 PMC3640615

[14] Carpenter JS Gautam S Freedman RR Andrykowski M. Circadian rhythm of objectively recorded hot flashes in postmenopausal breast cancer survivors. Menopause. (2001) 8:181–8. 10.1097/00042192-200105000-00007 11355040

[15] Freedman RR. Hot flashes revisited. Menopause. (2000) 7:3–4. 10646697

[16] Freedman RR Norton D Woodward S Cornélissen G. Core body temperature and circadian rhythm of hot flashes in menopausal women. J Clin Endocrinol Metab. (1995) 80:2354–8. 10.1210/jcem.80.8.7629229 7629229

[17] Freedman RR Krell W. Reduced thermoregulatory null zone in postmenopausal women with hot flashes. Am J Obstet Gynecol. (1999) 181:66–70. 10.1016/s0002-9378(99)70437-0 10411797

[18] Casper RF Yen SS. Neuroendocrinology of menopausal flushes: An hypothesis of flush mechanism. Clinical Endocrinology. (1985) 22:293–312. 10.1111/j.1365-1985tb03243.x 3884189

[19] Sturdee DW. The menopausal hot flush—Anything new? Maturitas. (2008) 60:42–9. 10.1016/j.maturitas.02006 18384981

[20] Freeman DEW Sherif K. Prevalence of hot flushes and night sweats around the world: a systematic review. Climacteric. (2007) 10:197–214. 10.1080/13697130601181486 17487647

[21] Thurston RC Bromberger JT Joffe H Avis NE Hess R Crandall CJ . Beyond frequency: who is most bothered by vasomotor symptoms?. Menopause (New York, NY). (2008) 15:841–7. 10.1097/gme.0b013e318168f09b 18521049 PMC2866103

[22] Stein KD Jacobsen PB Hann DM Greenberg H Lyman G. Impact of hot flashes on quality of life among postmenopausal women being treated for breast cancer. J Pain Symp Manage. (2000) 19:436–45. 10.1016/S0885-3924(00)00142-1 10908824

[23] Clemons M Goss P. Estrogen and the risk of breast cancer. N Eng J Med. (2001) 344:276–85. 10.1056/NEJM200101253440407 11172156

[24] Velie EM Nechuta S Osuch JR. Lifetime reproductive and anthropometric risk factors for breast cancer in postmenopausal women. Breast Dis. (2006) 24:17–35. 10.3233/BD-2006-24103 16917137

[25] Rossouw JE Anderson GL Prentice RL LaCroix AZ Kooperberg C Stefanick ML . Risks and benefits of estrogen plus progestin in healthy postmenopausal women: principal results from the women's health initiative randomized controlled trial. JAMA. (2002) 288:321–33. 10.1001/jama.288.3.321 12117397

[26] Neff MJ. NAMS releases position statement on the treatment of vasomotor symptoms associated with menopause. Am Fam Physician. (2004) 70:393. 15291096

[27] Elkins G Marcus J Stearns V Perfect M Rajab MH Ruud C . Randomized trial of a hypnosis intervention for treatment of hot flashes among breast cancer survivors. J Clin Oncol. (2008) 26:5022–6. 10.1200/JCO.166389 18809612 PMC2652097

[28] Elkins G Marcus J Stearns V Hasan Rajab M. Pilot evaluation of hypnosis for the treatment of hot flashes in breast cancer survivors. Psychooncology. (2007) 16:487–92. 10.1002/pon.1096 17048223

[29] Elkins GR Fisher WI Johnson AK Carpenter JS Keith TZ. Clinical hypnosis in the treatment of postmenopausal hot flashes: A randomized controlled trial. Menopause. (2013) 20:291–8. 10.1097/gme.0b013e31826ce3ed 23435026 PMC3556367

[30] Handley A Williams M. The efficacy and tolerability of SSRI/SNRIs in the treatment of vasomotor symptoms in menopausal women: a systematic review. J Am Assoc Nurse Pract. (2015) 27:54–61. 10.1002/2327-6924.12137 24944075

[31] Noven Pharmaceuticals Inc. First and only FDA-approved, non-hormonal treatment for moderate to severe hot flashes now available by prescription in U.S. pharmacies [Press release]. (2013). Available online at: http://www.noven.com/PR%20PDFs/2013%20PR%20PDFs/PR110513.pdf

[32] Orleans RJ Li L Kim M Guo J Sobhan M Soule L . FDA approval of paroxetine for menopausal hot flushes. N Engl J Med. (2014) 370:1777–9. 10.1056/NEJMp1402080 24806158

[33] Archer DF Sturdee DW Baber R de Villiers TJ Pines A Freedman RR . Menopausal hot flushes and night sweats: where are we now? Clim J Int Menop Soc. (2011) 14:515–28. 10.3109/13697137.2011.608596 21848495

[34] Shanafelt TD Barton DL Adjei AA Loprinzi CL. Pathophysiology and treatment of hot flashes. Mayo Clinic Proceed. (2002) 77:1207–18. 10.4065/77.11.1207 12440557

[35] Fantasia HC. A nonhormonal treatment for moderate to severe vasomotor symptoms of menopause. Nursing for Women's Health. (2016) 20:511–8. 10.1016/j.nwh.08007 27719781

[36] Noven Pharmaceuticals Inc. Paroxetine: Highlights of Prescribing Information. Miami, FL: Author (2013).

[37] Aursnes I Tvete IF Gaasemyr J Natvig B. Suicide attempts in clinical trials with paroxetine randomised against placebo. BMC Med. (2005) 3:14. 10.1186/1741-7015-3-14 16115311 PMC1198229

[38] Benedetti F. Placebo effects: From the neurobiological paradigm to translational implications. Neuron. (2014) 84:623–37. 10.1016/j.neuron.10023 25442940

[39] Kirsch I. Placebo: The role of expectancies in the generation and alleviation of illness. In P. Halligan and A. Mansel (Eds.), The Power of Belief: Psychosocial Influence on Illness, Disability and Medicine Oxford: Oxford University Press (2006) (pp. 55-67).

[40] Kirsch I. Response expectancy as a determinant of experience and behavior. Am Psychol. (1985) 40:1189–202. 10.1037/0003-066X.40.11.1189

[41] Kirsch I. Chapter five—Response expectancy and the placebo effect. In L. Colloca (Ed.), *International Review of Neurobiology*. Academic Press. (2018) (Vol. 138, pp. 81–93). 10.1016/bs.irn.01003 29681336

[42] Kirsch I Sapirstein G. Listening to Prozac but hearing placebo: a meta-analysis of antidepressant medication. Prevent Treat. (1998) 1:12. 10.1037./1522-3736.1.1.12a

[43] Kirsch I Moore T Scoboria A Nicholls S. The emperor's new drugs: an analysis of antidepressant medication data submitted to the U.S. food and drug administration. Prevent Treat. (2002) 5:23. 10.1037./1522-3736.5.1.523A

[44] Kirsch I Deacon BJ Huedo-Medina TB Scoboria A Moore TJ Johnson BT . Initial severity and antidepressant benefits: a meta-analysis of data submitted to the food and drug administration. PLoS Med. (2008) 5:e45. 10.1371/journal.pmed.0050045 18303940 PMC2253608

[45] Fountoulakis KN Möller HJ. Efficacy of antidepressants: a re-analysis and re-interpretation of the Kirsch data. Int J Neuropsychopharmacol. (2011) 14:405–12. 10.1017/S1461145710000957 20800012

[46] Fournier JC DeRubeis RJ Hollon SD Dimidjian S Amsterdam JD Shelton RC . Antidepressant drug effects and depression severity: a patient-level meta-analysis. JAMA. (2010) 303:47–53. 10.1001/jama.2009.1943 20051569 PMC3712503

[47] Turner EH Matthews AM Linardatos E Tell RA Rosenthal R. Selective publication of antidepressant trials and its influence on apparent efficacy. N Eng J Med. (2008) 358:252–60. 10.1056/NEJMsa065779 18199864

[48] Colloca L Klinger R Flor H Bingel U. (Placebo analgesia: Psychological and neurobiological mechanisms. Pain. (2013) 154:511–514. 10.1016/j.pain.02002 23473783 PMC3626115

[49] Kirsch I. Placebo effect in the treatment of depression and anxiety. Front Psychiatry. (2019) 10:407. 10.3389./fpsyt.2019.00407 31249537 PMC6584108

[50] Kaptchuk TJ Friedlander E Kelley JM Sanchez MN Kokkotou E Singer JP . Placebos without deception: a randomized controlled trial in irritable bowel syndrome. PLoS ONE. (2010) 5:e15591. 10.1371/journal.pone.0015591 21203519 PMC3008733

[51] Araujo ACD Silva FGD Salvi F Awad MC Silva EAD Damião R . The management of erectile dysfunction with placebo only: does it work? J Sex Med. (2009) 6:3440–8. 10.1111/j.1743-6109.2009.01496.x 19758285

[52] Maclennan AH Broadbent JL Lester S Moore V. Oral oestrogen and combined oestrogen/progestogen therapy versus placebo for hot flushes. Cochrane Database Syst. Rev. (2004) 2004:CD002978. 10.1002/14651858.CD002978.pub2 15495039 PMC7004247

[53] Li L Xu L Wu J Dong L Lv Y Zheng Q . Quantitative analysis of placebo response and factors associated with menopausal hot flashes. Menopause. (2017) 24:932–7. 10.1097/GME.0000000000000858 28399006

[54] Simon JA Portman DJ Kaunitz AM Mekonnen H Kazempour K Bhaskar S . Low-dose paroxetine 7.5 mg for menopausal vasomotor symptoms: tTwo randomized controlled trials. Menopause. (2013) 20:1027–35. 10.1097/GME.0b013e3182a66aa7 24045678

[55] Stearns V Slack R Greep N Henry-Tilman R Osborne M Bunnell C . Paroxetine is an effective treatment for hot flashes: Results from a prospective randomized clinical trial. J Clin Oncol. (2005) 23:6919–6930. 10.1200/JCO.10081 16192581

[56] Capriglione S Plotti F Montera R Luvero D Lopez S Scaletta G . Role of paroxetine in the management of hot flashes in gynecological cancer survivors: Results of the first randomized single-center controlled trial. Gynecol Oncol. (2016) 143:584–8. 10.1016/j.ygyno.10006 27751589

[57] Stearns V Beebe KL Iyengar M Dube E. Paroxetine controlled release in the treatment of menopausal hot flashes: a randomized controlled trial. JAMA. (2003) 289:2827–34. 10.1001/jama.289.21.2827 12783913

[58] Noven Therapeutics. A phase 2, exploratory, eight-week, multicenter, double-blind, randomized, placebo-controlled, efficacy and safety study of mesafem (Paroxetine Mesylate) capsules in the treatment of vasomotor symptoms associated with menopause (Clinical Trial Registration results/NCT00786188). clinicaltrials.gov. (2015). Available online at: https://http://clinicaltrials.gov/ct2/show/results/NCT00786188 (accessed March 29, 2023).

[59] Simon JA Chandler J Gottesdiener K Lazarus N He W Rosenberg E . Diary of hot flashes reported upon occurrence: Results of a randomized double-blind study of raloxifene, placebo, and paroxetine. Menopause. (2014) 21:938–44. 10.1097/GME.0000000000000218 24569618

[60] Carroll DG Lisenby KM Carter TL. Critical appraisal of paroxetine for the treatment of vasomotor symptoms. Int J Women's Health. (2015) 7:615–24. 10.2147/IJWH.S50804 26124682 PMC4476484

[61] Greenberg RP Bornstein RF Zborowski MJ Fisher S Greenberg MD. A meta-analysis of fluoxetine outcome in the treatment of depression. J Nerv Ment Dis. (1994) 182:547–51. 10.1097/00005053-199410000-00003 7931201

[62] Riemma G Schiattarella A La Verde M Zarobbi G Garzon S Cucinella G . Efficacy of low-dose paroxetine for the treatment of hot flushes in surgical and physiological postmenopausal women: systematic review and meta-analysis of randomized trials. Medicina. (2019) 55:554. 10.3390./medicina55090554 31480427 PMC6780738

[63] Wei D Chen Y Wu C Wu Q Yao L Wang Q . Effect and safety of paroxetine for vasomotor symptoms: Systematic review and meta-analysis. BJOG Int J Obstet Gynaecol. (2016) 123:1735–43. 10.1111/1471-0528.13951 27062457

